# Nicotinamide mononucleotide adenylyltransferase of *Trypanosoma cruzi* (TcNMNAT): a cytosol protein target for serine kinases

**DOI:** 10.1590/0074-02760160103

**Published:** 2016-10-24

**Authors:** Diana Milena Sánchez-Lancheros, Luis Fernando Ospina-Giraldo, María Helena Ramírez-Hernández

**Affiliations:** 1 Universidad Nacional de Colombia, Departamento de Biología, Bogotá, Colombia; 2 Universidad Nacional de Colombia, Departamento de Farmacia, Bogotá, Colombia

**Keywords:** Trypanosoma cruzi, NAD^+^ biosynthesis, NMNAT, polyclonal antibodies, subcellular location, post-transcriptional modifications

## Abstract

Nicotinamide/nicotinate adenine dinucleotide (NAD+/NaAD) performs essential functions in cell metabolism and energy production due to its redox properties. The nicotinamide/nicotinate mononucleotide adenylyltransferase (NMNAT, EC 2.7.7.1/18) enzyme catalyses the key step in the biosynthesis of NAD+. Previously, the enzyme NMNAT was identified in *Trypanosoma cruzi* (TcNMNAT), a pathogenic agent with epidemiological importance in Latin America. To continue with the functional characterisation of this enzyme, its subcellular location and its possible post-translational modifications were examined in this study. For this, polyclonal antibodies were generated in mice, with soluble and denatured recombinant protein being used to detect the parasite’s NMNAT. Immunodetection assays were performed on whole extracts of *T. cruzi*, and an approximation of its intracellular location was determined using confocal microscopy on wild and transgenic parasites, which revealed the cytosol distribution patterns. This localisation occurs according to the needs of the dinucleotides that exist in this compartment. Additionally, a bioinformatics study was performed as a first approach to establish the post-translational modifications of the enzyme. Possible phosphorylation events were experimentally analysed by western blot, highlighting TcNMNAT as a potential target for serine kinases.

*Trypanosoma cruzi* is the protozoan parasite that causes American trypanosomiasis, which is also known as Chagas disease. According to calculations, approximately 16 to 18 million people are infected, and 120 million are at risk of becoming infected. The available medicaments, benznidazole and nifurtimox, are not entirely effective and cause multiple secondary effects. Moreover, due to their long administration periods, parasite resistance has developed (Dias & Schofield 2010).

Energy metabolism in protozoan pathogen parasites such as the trypanosomatids has not been extensively studied. Adenine and nicotinamide dinucleotide (NAD+) is widely known as a redox coenzyme and serves as a substrate in mono/poly ADP ribosylation reactions and in the synthesis of Ca^2+^-mobilising molecules, such as cyclic ADP-ribose and nicotinate and adenine dinucleotide phosphate (NAADP). Given the importance of NAD+ in energy metabolism and in cell signaling, the existence of several biosynthesis pathways is no surprise. These pathways converge in the step catalysed by the enzyme nicotinamide/nicotinate mononucleotide adenylyltransferase NMNAT (EC 2.7.7.1/18) (Berger et al. 2004).

Previously, our group identified the enzyme NMNAT in *T. cruzi* (Niño et al. 2015). This study is presented as an approach to its localisation to establish similarities and differences with its human orthologs, with the aim to gain a better understanding of the host-parasite relation and to enable the development of new tools to fight these parasitic infections.

## MATERIALS AND METHODS

*Prokaryotic expression and purification of the recombinant His-TcNMNAT protein* - The TcNMNAT protein, fused to a histidine tag, was expressed and purified from the soluble fraction after cellular lysis, using nickel affinity chromatography. The purification was monitored by SDS-PAGE as described previously (Niño et al. 2015). On the other hand, the inclusion body was solubilised using reported protocols (Sambrook & Rusell 2001). Later, the solubilised protein from inclusion bodies was purified using preparative SDS-PAGE (Mohammadian et al. 2010).

*Production of anti-His-TcNMNAT polyclonal antibodies, generated against the protein from inclusion bodies and against the protein purified from soluble fraction* - This step was performed using previously standardised protocols (Harlow & Lane 1988), wherein 50 µg of recombinant protein was used to perform four inoculations in *Mus musculus* BALB-C mice. Blood collection was performed every eight days after the inoculations were performed. The antibodies produced were evaluated using an ELISA and a titer of 1:10000 was determined (Moreno-González et al. 2013); the antibodies were purified using affinity from western blot (Fang 2012).

*T. cruzi parasite culture* - Epimastigote forms of the CL Brener *T. cruzi* strain were cultured in vitro at 27ºC using Schenider’s Insect Medium at pH 6.9, sterilised by filtration and supplemented with 10% fetal bovine serum (Campos et al. 2009). Approximately, 1x10^7^ parasites (counted in a Neubauer chamber) were collected in logarithmic growth phase.

*Immunodetection assays in T. cruzi extracts* - The complete washed parasites were resuspended in protein loading buffer, and DTT was added. Approximately 1x10^6^ parasites were loaded per SDS-PAGE well for use in western blots on nitrocellulose membranes (Thermo). A 1:1000 dilution of the antibody produced was used, and as a secondary antibody, the anti-mouse-peroxidase bound antibody was used (Sigma). The revealing step was performed with 4-chloronaphthol (Promega) (Walker 2002).

*Immunoprecipitation of the Tc-NMNAT protein* - The parasites (5x10^8^) were incubated in lysis buffer (0.1X PBS, 0.1% Triton X-100), protease inhibitor cocktail (Sigma) and 1 mM Na_3_VO_4_, followed by 10 freeze-thawing cycles. After centrifugation at 12,000 × g for 20 min at 4ºC, the soluble fraction was supplemented with SDS at a final concentration of 0.2% w/v and with immunoprecipitation buffer (50 mM Tris-HCl, pH 7.5). This mix was heated at 95ºC for 10 min, and Triton X-100 was added at a final concentration of 0.5% v/v. Immunoprecipitation beads of protein A (GE Healthcare) were added to the sample and left for 1 h, followed by the extraction of the supernatant (clear extract). Each clear extract was supplemented with 10 µL of the corresponding antibody and agitated overnight at 4ºC. An incubation solution with beads was added, and the samples were incubated for 3 h on ice. The beads were washed four times with immunoprecipitation buffer, resuspended in protein loading buffer and heated at 95ºC for 10 min, followed by analysis with SDS-PAGE gels stained with silver (Walker 2002).

*Confocal microscopy in T. cruzi* - The parasites (2x10^5^) in epimastigote form were fixed with 4% (W/V) paraformaldehyde for 1 h at 4ºC, followed by a treatment with 100 mM glycine for 15 min. Cells were permeabilised with acetone for 5 min at 4ºC. The solution was blocked using 1% (W/V) BSA in PBS for 1 h. The samples were then incubated with the primary antibody (1:1000) for 1 h. The incubation with the secondary anti-IgG antibody bound to Alexa Fluor 488 (Abcam) was performed for 1 h in darkness. DNA labelling was performed with DAPI (1:7000) for 5 min in darkness. The slides were covered with Fluoromount mounting medium. The slides were observed under a Nikon C1 Plus ECLIPSE Ti confocal microscope and were analysed using the NIS elements AR software, with a 100X objective, a z of 2, a 480-nm detector laser 515/30 and a 488-nm detector laser 590/50 (Johndrow et al. 2014).

*Construction of the vector pTEX-TcNMNAT and T. cruzi epimastigote transfection* - In order to increase the NMNAT levels in the parasite and get clearer signals, we cloned the TcNMNAT coding sequence in the pTEX vector, a *T. cruzi* expression construct. Polymerase chain reaction (PCR) amplification was performed using the plasmid TcNMNAT-pET100, which has been previously described previously (Niño et al. 2015), as the template. The primers used contained a restriction site for BamHI in the 5′ end (5′-CGG GAT CCC GAT GAG CGA TGA CAC AT-3′) and a restriction site for EcoRI in the 3′ end (5′-CCG GAA TTC CGG TCA ACA ATT TTG AGT ATT-3′). Thirty cycles of 94ºC for 30 s, 50ºC for 30 s and 72ºC for 45 s were performed. A final extension was performed at 72ºC for 10 min. The PCR product of 896 bp was purified using a Wizard® PCR Clean-Up System kit, and the purified product was used to perform subcloning in the pGEM®-T Easy Vector System according to the manufacturer’s instructions (Promega). Both the pTEX vector and the TcNMNAT-pGEM®-T Easy vector were digested with EcoRI and BamHI (Fermentas). These products were purified and ligated to obtain the final construct (Sambrook & Rusell 2001), which was verified by sequencing.

For the electroporation, 5x10^7^ parasites were centrifuged at 1000 × g for 10 min at 4ºC, resuspended in sterile electroporation buffer (137 mM NaCl, 5 mM KCl, 5.5 mM Na_2_HPO_4_, 0.77 mM glucose, 21 mM HEPES, pH 7.2) and mixed with 30 µg of the plasmid. The electroporation was performed with two consecutive pulses at 350 V and 500 µF (Manque et al. 2003). Immediately, the electroporated parasites were transferred to a culture medium and after 24 h, G418 was added (Thermo) at a final concentration of 0.6 µg/µL.

*Phosphorylation study of the TcNMNAT protein* - Immunoprecipitations were performed as previously described, and commercial antibodies were used against amino acids (aas) 12-40 of the human isoenzyme 2 NMNAT protein produced in rabbits (Abcam) titer 1:200, monoclonal anti-S-phosphorylated produced in mice (Sigma) titer 1:1600, monoclonal anti-Y-phosphorylated produced in mice (Sigma) titer 1:4000 and polyclonal anti-T-phosphorylated produced in rabbits (Cell Signaling) titer 1:2000 (Papavassiliou 1994).

## RESULTS AND DISCUSSION

**Fig. 1:**
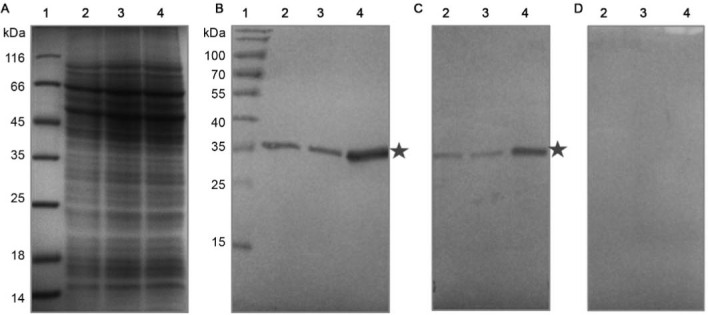
: recognition of the endogenous and overexpressed TcNMNAT protein in cellular extracts. (A) Discontinuous SDS-PAGE T12, Coomassie blue staining; (B-D) western blot, α- IgG-peroxidase, substrate 4-chloronaphthol, using the primary antibody; (B) αHis-TcNMNAT generated against the inclusion bodies 1:1000; (C) αHis-TcNMNAT generated against the native protein 1:300; (D) Pre-immune serum. (1) Pre-stained molecular weight marker; (2) Native *Trypanosoma cruzi*; (3) *T. cruzi* transfected with the empty pTEX vector; (4) *T. cruzi* transfected with the TcNMNAT–pTEX vector; stars: TcNMNAT.

To determine the location of the TcNMNAT protein, immunodetection and overexpression were used. Therefore, polyclonal antibodies were produced from a soluble and a denatured protein. Western blot assays were performed to study the TcNMNAT protein in the transfected parasites and nontransfected control. Fig. 1 shows the recognition of TcNMNAT by the generated and purified polyclonal antibodies. In Fig. 1A, no differences were observed in the electrophoresis profile among the samples analysed. Fig. 1B-D shows the immunodetection assays performed with the generated antibodies. A 35-kDa band is observed, which corresponds to the endogenous NMNAT in the extracts of the nontransfected and transfected cells with the empty vector. This band is more evident in cells transfected with the TcNMNAT-pTEX vector, confirming the identity of the TcNMNAT protein. The results obtained are similar using both generated antibodies. This band shows a higher size compared to the predicted molecular weight using bioinformatic tools (32 kDa). This increase can be due to possible post-translational modifications.

Five phosphorylation sites were predicted for S, two for T and one for Y from the analysis of the TcNMNAT protein sequence in the NetPhos 2.0 server (Blom et al. 1999). The analysis of the phosphoproteome of *T. cruzi* (Marchini et al. 2011) revealed the presence of 84.1% of the phosphorylated residues in S, 14.9% in T and 1.0% in Y, despite the fact that there are no genes encoding for tyrosine kinase enzymes in the *T. cruzi* genome.

**Fig. 2:**
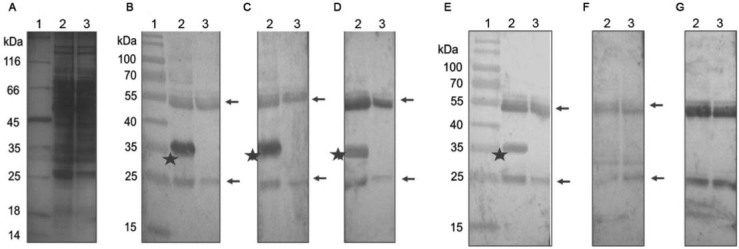
: immunoprecipitation using αHis-TcNMNAT generated against the inclusion bodies in *Trypanosoma cruzi* transfected with TcNMNAT-pTEX. (A) Discontinuous SDS-PAGE T12, silver staining; (B-G) western blot, α- IgG-Biotin/Streptavidin-alkaline phosphatase, substrate NBT/BCIP using the primary antibody; (B) αHis-TcNMNAT generated against the inclusion bodies 1:1000; (C) αHis-TcNMNAT generated against the soluble protein 1:300; (D) Phosphorylated-αS 1:1600; (E) αHsNMNAT-2 1:200; (F) Phosphorylated-αT 1:2000; (G) Phosphorylated-αY 1:4000. (1) Pre-stained molecular weight marker; (2) immunoprecipitation of *T. cruzi*; (3) control immunoprecipitation; stars: immunoprecipitated, overexpressed TcNMNAT; arrows: heavy and light chains of the antibodies.

For the analysis of the possible phosphorylation of the TcNMNAT protein, immunoprecipitations were performed on *T. cruzi* extracts using the antibodies generated. Such immunoprecipitates were analysed by western blot using commercial antibodies against the Y, S and T phosphorylated aas (Fig. 2).

Fig. 2 shows the result of the immunoprecipitation using the parasites transfected with vector TcNMNAT-pTEX. In the analysis of the results from the western blot, in Fig. 2B, the immunoprecipitation of a 35-kDa band is evident. This band is detected again with the antibody generated against the native protein and with a commercial antibody against the human NMNAT-2, thereby confirming its identity (Fig. 2C, E). TcNMNAT protein shows 65% of identity with the epitope recognised by the commercial antibody. This sequences are nucleotide binding motifs, characteristic of NMNAT proteins.

Regarding the phosphorylation, the antibody against the phosphorylated S also recognises the 35-kDa band (Fig. 2D), which was not detected by the antibodies against the phosphorylated T or Y (Fig. 2F-G).

This type of post-translational modification could be regulating the activity of the NMNAT protein, its interaction with other proteins or its subcellular localisation (Peck 2006). The human isoenzyme NMNAT-1 that is located in the nucleus shows phosphorylation in the S136 residue. This modification does not affect the isoenyme’s subcellular location but regulates its interaction with the PARP-1 protein (the enzyme that has the highest NAD+ consumption as substrate). When these two proteins associate, PARP-1 suffers a self-modification that increases its catalytic activity. The phosphorylated NMNAT enzyme does not bind to the PARP-1 protein, and therefore, the PARP-1 protein is not active. Therefore, the NMNAT protein not only provides the substrate for PARP-1 but also regulates its catalytic activity according to its phosphorylation state (Berger et al. 2007).

Once the generated antibodies were confirmed to recognise the NMNAT protein in situ, we performed a confocal microscopy assay to obtain a close-up view of the subcellular location of the protein using both of the generated antibodies.

**Fig. 3:**
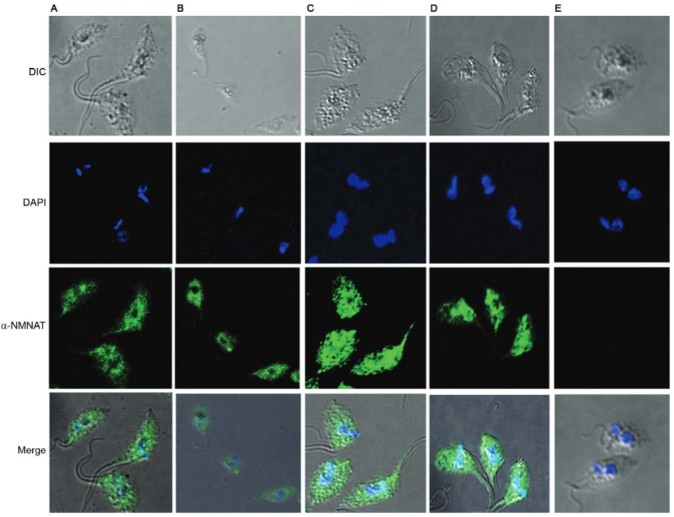
: location of the endogenous TcNMNAT protein in *Trypanosoma cruzi* epimastigotes using antibodies generated against the soluble protein. (A) Wild *T. cruzi*, IgG αHis-TcNMNAT 1:250; (B) *T. cruzi* transfected with the pTEX vector, IgG αHis-TcNMNAT 1:250; (C) *T. cruzi* transfected with the TcNMNAT-pTEX vector, IgG αHis-TcNMNAT 1:250; (D) *T. cruzi* transfected with the TcNMNAT-pTEX vector, pre-immune serum.

The first row of Fig. 3 shows the bright field image of the parasite in its epimastigote form. In the second (DAPI), two blue dots can be observed per parasite: one corresponding to the nuclear genetic material and the other to the kinetoplast. The third row (α-NMNAT) shows the signal emitted by the α-IgG coupled to the Alexa Fluor 488, revealing that this protein is not nuclear and that it shows particulate patterns of cytosolic distribution in the wild parasites (Fig. 3A).

When the experiment was performed using the parasites transfected with the empty vector (Fig. 3B), the same distribution pattern was observed as in the wild parasites. This was expected because the empty vector does not contain elements that change the characteristics of the protein under study. When the parasites that overexpress the TcNMNAT protein (Fig. 3C) and the antibody generated against the soluble protein were used, the location was verified with higher intensity because of the overexpression of the protein. Fig. 3D shows the pre-immune control, which does not show any recognition.

**Fig. 4:**
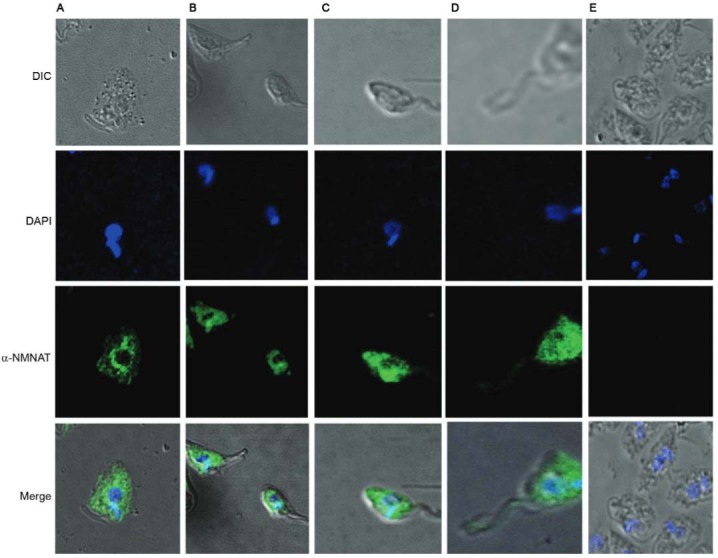
: location of the endogenous TcNMNAT protein in *Trypanosoma cruzi* epimastigotes using antibodies generated against the inclusion bodies. (A) Wild *T. cruzi* IgG αHis-TcNMNAT 1:500; (B) *T. cruzi* transfected with the pTEX vector, IgG αHis-TcNMNAT 1:500; (C) *T. cruzi* transfected with the TcNMNAT-pTEX vector, IgG αHis-TcNMNAT 1:500; (D) *T. cruzi* transfected with the TcNMNAT-pTEX vector, non-related serum.

When the same analysis was performed with the antibodies developed from the inclusion bodies, the cytoplasmic location could be seen again (Fig. 4). This indicates that this antibody is also capable of recognising the protein in its wild state, possibly due to the epitopes on its surface.

In parasites transfected with the vector TcNMNAT-pTEX, the location of the protein was confirmed with the higher intensity, which could be attributed to the amount of fluorescence emitted due to the protein overexpression.

In the cytosol, the synthesis of the calcium-mobilising molecules, such as NAADP, occurs along with the NAD+-dependent deacetylation of proteins by sirtuins such as TcSir2rpl, an enzyme involved in the proliferation of the replication forms of the parasite and in life-cycle differentiation, among other things (Sacconnay et al. 2014, Ritagliati et al. 2015). These processes require a constant supply of NAD+, explaining the presence of this enzyme in the cytosol. This observation was obtained by both immunodetection and overexpression of the transgenes.

The particulated/dotted distribution could be indicating the association of the enzyme with organelles. For example, human NMNAT isoenzyme 2 (HsNMNAT-2() has been located in cytosol and Golgi apparatus, depending on the cytosolic NAD concentration (Lau et al. 2010).

The endogenous NMNAT protein of *T. cruzi* has an approximate size of 35 kDa in wild and transgenic parasites, compared to the estimated 32 kDa, a difference that can be attributed to possible post-translational modifications. Under the conditions analysed, the NMNAT enzyme of *T. cruzi* most likely shows phosphorylation in one or some serine residues. This signal was observed in the immunoprecipitates of the native and transfected parasites.

*T. cruzi’*s NMNAT location has been determined as cytosolic, which agrees with the NAD demand in this cell region. The result was obtained by means of immunofluorescence, using antibodies generated against the recombinant protein purified from soluble fraction and inclusion bodies. It is possible that this enzyme is subject to post-translational modifications such as phosphorylations in serine residues.

## References

[B1] Berger F, Lau C, Ziegler M (2007). Regulation of poly(ADP-ribose) polymerase 1 activity by the
phosphorylation state of the nuclear NAD biosynthetic enzyme NMN adenylyl
transferase 1. Proc Natl Acad Sci USA.

[B2] Berger F, Ramírez-Hernández M, Ziegler M (2004). The new life of a centenarian: signalling functions of
NAD(P). Trends Biochem Sci.

[B3] Blom N, Gammeltoft S, Brunak S (1999). Sequence and structure-based prediction of eukaryotic protein
phosphorylation sites. J Mol Biol.

[B4] Campos Y, Briceño L, Reina K, Figarella K, Pérez J, Mosca W (2009). Serological diagnosis of Chagas disease: evaluation and
characterization of a low cost antigen with high sensitivity and
specificity. Mem Inst Oswaldo Cruz.

[B5] Dias JCP, Schofield CJ, Telleria J, Tibayrenc M (2010). Social and medical aspects: morbidity and mortality in
general population. American Trypanosomiasis.

[B6] Fang L (2012). Antibody purification from western blotting. Bio-Protocol.

[B7] Harlow E, Lane D (1988). Antibodies, a laboratory manual.

[B8] Johndrow C, Nelson R, Tanowitz H, Weiss L, Nagajyothi F (2014). Trypanosoma cruzi infection results in an increase in intracellular
cholesterol. Microbes Infect.

[B9] Lau C, Dölle C, Gossmann T, Agledal L, Niere M, Ziegler M (2010). Isoform-specific targeting and interaction domains in human
nicotinamide mononucleotide adenylyltransferases. J Biol Chem.

[B10] Manque PM, Neira I, Atayde VD, Cordero E, Ferreira AT, da Silveira JF (2003). Cell adhesion and Ca2+ signaling activity in stably transfected
Trypanosoma cruzi epimastigotes expressing the metacyclic stage-specific surface
molecule gp82. Infect Immun.

[B11] Marchini F, de Godoy L, Rampazzo R, Pavoni D, Probst C, Gnad F (2011). Profiling the Trypanosoma cruzi phosphoproteome. PLoS ONE.

[B12] Mohammadian T, Doosti M, Paknejad M, Siavoshi F, Massarrat S (2010). Preparative SDS-PAGE electroelution for rapid purification of alkyl
hydroperoxide reductase from Helicobacter pylori. Iran J Public Health.

[B13] Moreno-González PA, Díaz-González GJ, Ramírez-Hernández M (2013). Production and purification of avian antibodies (IgYs) from inclusion
bodies of a recombinant protein central in NAD+ metabolism. Rev Colomb Quim.

[B14] Niño CH, Forero-Baena N, Contreras LE, Sánchez-Lancheros D, Figarella K, Ramírez MH (2015). Identification of the nicotinamide mononucleotide adenylyltransferase
of Trypanosoma cruzi. Mem Inst Oswaldo Cruz.

[B15] Papavassiliou AG (1994). Preservation of protein phosphoryl groups in immunoprecipitation
assays. J Immunol Methods.

[B16] Peck SC (2006). Analysis of protein phosphorylation: methods and strategies for
studying kinases and substrates. Plant J.

[B17] Ritagliati C, Alonso VL, Manarin R, Cribb P, Serra EC (2015). Overexpression of cytoplasmic TcSIR2RP1 and mitochondrial TcSIR2RP3
impacts on Trypanosoma cruzi growth and cell invasion. PLoS Negl Trop Dis.

[B18] Sacconnay L, Angleviel M, Randazzo G, Queiroz M, Queiroz E, Wolfender JL (2014). Computational studies on sirtuins from Trypanosoma cruzi: structures,
conformations and interactions with phytochemicals. PLoS Negl Trop Dis.

[B19] Sambrook J, Rusell D (2001). Molecular cloning: a laboratory manual.

[B20] Walker JM (2002). The protein protocols handbook.

